# CD36 as a marker of acute myeloid leukemia prognosis: A systematic review

**DOI:** 10.1016/j.htct.2025.103861

**Published:** 2025-06-16

**Authors:** Marina Chaves Amantéa, Rafaela Pires da Silva, Larissa Ranini Soares, João Lorenzo de Medeiros Pereira, Ana Paula Duarte de Souza

**Affiliations:** Escola de Ciências da Saúde e da Vida, Pontifícia Universidade Católica do Rio Grande do Sul (PUCRS), Porto Alegre, RS, Brazil

**Keywords:** Acute myeloid leukemia, Antigen, Cd36, Prognosis, Prognostic factors

## Abstract

CD36 is a glycoprotein associated with resistance to chemotherapy and the recurrence of acute myeloid leukemia. This systematic review aims to evaluate the impact of CD36 on the prognosis of acute myeloid leukemia, a complex heterogeneous malignant hematopoietic disease. The Embase, Scopus, Web of Science, Cochrane Library and SciELO databases were searched until September 2023. Only studies that analyzed CD36 expression in humans were included. Of 905 articles identified from the databases, 600 were screened and nine were included. The Newcastle-Ottawa Scale was used to evaluate the methodological quality of the studies. According to this systematic review, CD36 is associated with different prognostic factors in acute myeloid leukemia, including remission and relapse of the disease, overall survival, and chemoresistance.

## Introduction

Acute myeloid leukemia (AML) is a heterogeneous group of fatal and aggressive diseases affecting hematopoietic stem cells. Mutations in myeloid stem cells occur during AML progression, leading to an immature cell population.^1^ Currently, the treatments for AML are chemotherapy and bone marrow transplantation; however, there is a need for new therapeutic approaches.^2^^,^^3^

Among leukemia subtypes, AML accounts for the highest percentage of leukemia-related deaths, representing 62 % of cases.^4^ AML is the second most common subtype in children,^5^ resulting in about 15–20 % of leukemia cases in this population.^6^ In developed countries, survival rates in pediatric patients are high (65 %) compared to low- and middle-income countries, where rates are lower than 40 %.^7^ AML relapse rates vary from 30–35 % in younger patients with favorable risk factors and can reach 80 % in older patients with adverse risk factors.^8^ The prognosis of AML patients is based on the absence or presence of cytogenetic and/or molecular biology abnormalities and is divided into favorable, intermediate, and unfavorable subgroups.^9^ Prognostic markers in AML include mutations in the *NPM1, FLT3, MLL*, and *CEBPα* genes, as well as alterations in the expression levels of *BAALC, MN1, ERG*, and *AF1q,*^10^ and chromosomal abnormalities such as t(8;21) and t(15;17).^11^ Yet, accurately predicting prognosis in AML remains challenging due to factors such as disease heterogeneity, clonal evolution, and the influence of microenvironmental factors. There is still debate about the influence of blast immunophenotyping on the prognosis in AML.^12^ Recent studies have shown worse prognoses for AML associated with increased CD34 and CD318 expressions.^13^^,^^14^ Also, CD36 is positively associated with the dissemination of leukemic blasts and is highly expressed in tumors at an advanced stage.^15^ Furthermore, leukemia cells resistant to cytarabine (Cytarabine) exhibit a high expression of CD36.^16^

CD36 is a glycoprotein of the scavenger receptor class B superfamily, the gene of which is located on the long arm of chromosome 7 (7q11.2).^17^ This glycoprotein has different physiological functions such as cell adhesion, establishment of connections with collagen, thrombospondin, phospholipids, and low-density lipoprotein, and can serve as a regulatory glycoprotein for fatty acid transport.^18^ CD36 has been described as contributing to tumor formation and the development of various types of cancer, including breast cancer, gastric cancer, and AML.^19^ CD36 expression is correlated with low survival in patients with lung carcinoma, bladder cancer, and luminal breast cancer.^20^^,^^21^ This study aims to review evidence of the association of CD36 with worse prognosis in AML.

## Material and methods

### Protocol

The protocol of this systematic review was registered *a priori* in the International Prospective Register of Systematic Reviews (PROSPERO) with registration number CRD42023481493.^22^

### Search

The search for articles was carried out by two reviewers, on September 16th, 2023, in six bibliographic databases: PubMed, Embase, Scopus, Web of Science, the Cochrane Library, and SciELO. The terms used were “acute myeloid leukemia” and all of its synonyms in MeSH Terms (or Emtree, in Embase) AND ‘CD36’ and all of its synonyms in MeSH Terms (or Emtree, in Embase). The complete search strategy can be accessed in the PROSPERO protocol.^22^ Additional research was also carried out using Google Scholar before extracting data, looking for new studies not yet peer-reviewed.

### Eligibility criteria

Studies that analyzed the expression of CD36 in human patients, including clinical trials, case-control studies, and case reports were included. The articles should evaluate the impact of CD36 on factors related to the prognosis of AML, including, for example, survival and response to treatment. Reviews, editorials, letters, abstracts from conference annals, and studies that only used animals or lineage cells for the analyses of CD36 were excluded. Studies that addressed only one specific subtype of AML and not the disease spectrum were also excluded.

### Study selection

Two independent reviewers selected studies based on eligibility criteria. Disagreements between individual judgments were solved by a third reviewer. The software used for blinding and recording decisions was Rayyan.^23^

### Data extraction

From each eligible study, data were input in duplicate in an Excel worksheet, following a standardized template created for this review. The following data were extracted from articles: first author, publication year, research location, study design, sample size, age of participants, percentage of males, eligibility criteria, stem cell source, method of CD36 quantification, comparators, statistical analysis, method of AML classification, evaluated prognostic factors and results related to the impact of CD36 on factors influencing the prognosis of AML.

### Quality assessment

The methodological quality of nonrandomized studies was evaluated using the Newcastle-Ottawa Scale. Studies rated 3–4 stars in the selection domain, 1–2 stars in the comparability domain and 2–3 stars in the outcome/exposure domain were classified as good quality. Studies rated 2 stars in the selection domain, 1–2 stars in the comparability domain and 2–3 stars in the outcome/exposure domain were considered as fair quality. Those rated 0–1 star in the selection domain or 0 stars in the comparability domain or 0–1 star in the outcome/exposure domain were classified as poor quality.^24^ Two independent reviewers evaluated the quality of the studies, and disagreements between individual judgements were solved by a third reviewer to reduce the risk of bias.

## Results

### Search results

**Figure 1:**
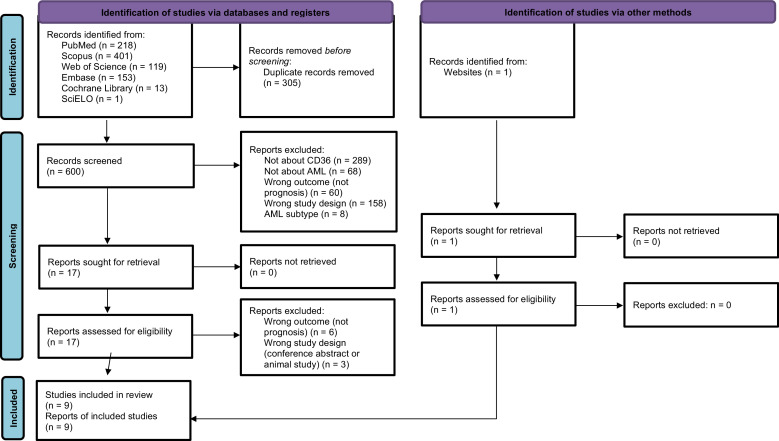
Flow diagram of study selection process.

The search strategy resulted in 905 articles, and one more article was added after a manual review to identify eligible articles that might not have been captured by the search strategy. After excluding 305 duplicates, the titles and abstracts of 600 articles remained were assessed and another 583 articles were excluded based on the eligibility criteria, leaving 17 articles and one added manually giving a total of 18 to be read in full. Of these, nine articles^25^, ^26^, ^27^, ^28^, ^29^, ^30^, ^31^, ^32^, ^33^ were included in this review with the other nine being excluded for not complying with the inclusion criteria (six did not report the prognoses, two were conference abstracts and one was exclusively an animal study). The agreement between evaluators during the full-text phase was 93.75 % (Cohen’s κ: 0.875). Figure 1 represents the flowchart detailing the excluded articles at each stage, along with the reasons for their exclusion.

### Study characteristics

Table 1 summarizes the characteristics of the studies included in the main analysis. Articles were published between 1992 and 2023. Some of the articles do not provide all the collected data, such as the mean age and gender of the study participants. From the studies that reported the mean age of the participants, the mean age ranged from more than one month to approximately 44 years. The stem cell source in most articles was the bone marrow. Two studies used peripheral blood in addition to bone marrow, and one of the articles used databases and did not clarify the stem cell source but mentioned the use of tumor tissue. The most commonly used method for CD36 quantification was flow cytometry, in addition to RNA sequencing and immunohistochemistry. The comparison was mainly conducted between patients with complete remission of AML versus patients who relapsed, patients with positive or increased CD36 expression versus negative or decreased CD36 expression, but there were also comparisons with healthy individuals and people with other types of cancer. The articles primarily classified AML based on the French-American-British (FAB) classification and Cytogenetic and Molecular Risk Groups. The studies demonstrate the impact of CD36 on the prognosis of AML across different categories, such as survival, remission, chemotherapy resistance, and tumor cell proliferation. All results linking CD36 expression to any of these characteristics were collected.

**Table 1:** Characteristics of studies included in the analysis.

First author	Country	Study quality	n	Stem cell source	Method of CD36 quantification	Classification of AML	Results
Smith, FO^25^	USA	Fair	176	BM	Flow cytometry	FAB	No prognostic significance for the cell surface expression of CD36 in myeloid cells.
Valet, G^31^	Germany	Fair	724	BM and PB	Flow cytometry	*C* + *M* Risk	5-year non-survivors had a higher percentage of CD36-positive AML blasts than survivors.
Perea, G^30^	Spain	Good	266	BM	Flow cytometry	FAB and *C* + *M* Risk	The 2-year LFS rate was lower in CD36+ patients (32 %) compared to CD36− patients (56 %; p-value = 0.01). The risk of relapse was higher in CD36+ patients (63 %) than in CD36− patients (38 %). CD36+ patients with trisomy 8 had poorer LFS (0 %) compared to CD36+ patients without trisomy 8 (37 %; *p*-value = 0.0001). Trisomy 8 was significantly associated with CD36 expression.
El-Aziz, A^29^	Egypt	Good	97	BM and PB	Flow cytometry, FISH	FAB	Remission was achieved in 23 of 45 (51.1 %) evaluable CD36+ patients and in 32 of 46 (69.5 %) CD36− patients. Although a higher number of responders was observed among CD36− patients compared to CD36+ patients, the difference was not statistically significant (*p*-value = 0.073). The 2-year OS rates were 37.5 % for CD36+ patients and 44.9 % for CD36− patients (p-value = 0.001). The 2-year LFS rates were 33.3 % for CD36+ patients and 40.6 % for CD36− patients (*p*-value = 0.03). CD36 expression, along with WBC count and adverse cytogenetics, were significant factors influencing OS and LFS. Multivariate analysis confirmed that CD36 expression retained its significance as an independent negative prognostic factor for both OS and LFS. CD36 expression was more commonly observed in the unfavorable cytogenetic group.
Zhang, T^33^	USA	Fair	196⁎	BM and PB	Flow cytometry	FAB and *C* + *M* Risk	Using the GSE30377 dataset (*n* = 23), patients were dichotomized based on the median CD36 expression. Patients with high CD36 expression exhibited shorter OS, although the difference was not statistically significant (*p*-value = 0.114). Analysis of the TCGA dataset (*n* = 173) showed that when patients were dichotomized by APOC2 and/or CD36 mRNA expression Z-scores into high (Z-score ≥ 2) and low (Z-score <2) groups, those with high APOC2 and/or CD36 expression had significantly shorter OS compared to patients with low APOC2 and low CD36 expression (median OS 9.2 vs. 21.5 months, *p*-value = 0.017).
Chen, YJ^27^	China	Low	70⁎	Database, undetermined (possibly tumor tissue)	RNA sequencing and immunohisto-chemistry	–	CD36 expression was significantly elevated in AML based on RNA-seq data. CD36 expression showed a strong positive correlation with infiltrating stromal scores in AML (*r* = 0.618, *p*-value <0.001). CD36 expression was positively correlated with immune scores (*r* = 0.609, *p*-value <0.001) and with the ESTIMATE score (*r* = 0.669, *p*-value <0.001), indicating a robust association between CD36 expression and the tumor immune microenvironment. CD36 expression was negatively correlated with the expression of four methyltransferases in AML
Hoch, REE^26^	Brazil	Good	51	BM	Flow cytometry	FAB and *C* + *M* Risk	Higher frequency of CD36+ cells at diagnosis was observed in cases with disease recurrence.
Zhang, Y^28^	China	Fair	5⁎	BM	Flow cytometry	–	CD36+ cells exhibited lower sensitivity to chemotherapeutics than CD36− cells.
Farge, T^32^	France	Fair	1273	BM and PB	Flow cytometry	*C* + *M* Risk	CD36 expression in blasts at diagnosis is associated with human AML progression and relapse. CD36 was significantly associated with a worse EFS (HR: 1.55; 95 % CI: 1.17–2.05, *p*-value = 0.002), and a worse OS (HR: 1.69; 95 % CI: 1.18–2.41, *p*-value = 0.005). Median EFS of CD36-high patients was half that of CD36-low patients (252 days vs. 538 days, respectively; HR:1.65, *p*-value <0.0001). Median OS, which was 462 days in CD36-high patients, was not reached after 3 years in CD36-low patients (HR: 1.88, *p*-value <0.0001). A high CD36 protein expression at diagnosis was associated with an increased CIR after intensive chemotherapy, and a high expression of CD36 was associated with a shorter CIR (SHR: 1.53; 95 % CI: 1.07–2.19, *p*-value = 0.02). Intermediate and unfavorable karyotypes had a higher percentage of CD36-expressing blasts. A comparison of the genomic landscape in 224 AML did not reveal a pattern related to CD36 expression, except for FLT3 abnormalities. there's an enrichment of AML with KMT2A (11q23) or t(9;22) abnormalities in CD36-high group and an increase in t(15;17) and t(8;21) AML in CD36-low group.

BM: bone marrow; PB: peripheral blood; LFS: leukemia-free survival; OS: overall survival; WBC: white blood cell; EFS: even-free survival; 95 % CI: 95 % confidence interval; CIR: cumulative incidence of relapse; FAB: French-American-British; *C* + *M* Risk: cytogenetic and molecular risk groups.

⁎ Sample size used to evaluate the outcome of interest.

### Quality assessment

The methodological quality of the studies analyzed in this review ranged from low to good, with most of the articles being classified as fair quality, primarily due to significant gaps in crucial information reported by the articles. Scores on the Newcastle-Ottawa Scale for the studies ranged from 5–7 stars.

### Synthesis of results

In a prospective study investigating the prognostic significance of cell surface antigens associated with myeloid differentiation, such as CD36, the authors found that the presence of these myeloid-associated cell surface antigens did not have prognostic significance for children.^25^ However, a higher frequency of CD36-positive cells at diagnosis was identified in cases of children who experienced disease recurrence.^26^ In contrast, in adults, CD36 expression was significantly higher in AML when compared to other types of cancer,^27^ and the increase or even the expression of CD36 is associated with several factors that appear to be associated with a poor prognosis. Bone marrow mononuclear cells from AML patients expressing CD36 are less susceptible to chemotherapeutic agents such as cytosine arabinoside (Ara-C) compared to cells that do not express CD36,^28^ demonstrating that CD36 expression interferes with chemotherapy. Another study also demonstrated a tendency for a lower response to chemotherapy in the group of patients with positive CD36 expression compared to the group with negative CD36 expression.^29^

Furthermore, CD36 expression is associated with relapse and recurrence of the disease. The higher frequency of CD36-positive cells at diagnosis was identified in cases that presented recurrence of the disease,^26^ as well as a higher risk of relapse for CD36^+^ patients, thereby presenting a shorter leukemia-free survival rate.^30^

In addition to a higher risk of relapse, CD36 expression is also associated with shorter overall survival. Five-year non-survivors showed increased levels of CD36-positive AML blasts,^31^ while complete remission was achieved in a higher percentage of CD36-nagative patients, as well as overall survival rates and leukemia-free survival rates.^29^

CD36 expression in blasts at diagnosis is also associated with human AML progression and relapse. CD36 was significantly associated with worse survival. The survival of CD36-high patients was half that of CD36-low patients. Furthermore, a high CD36 protein expression at diagnosis was associated with an increased cumulative incidence of relapse after intensive chemotherapy, and a multivariate analysis showed that a high expression of CD36 was associated with a shorter cumulative incidence of relapse.^32^

Some proteins may be associated with this CD36 effect, such as apolipoprotein C-II (APOC2), which cooperates with CD36 to promote leukemia growth, as described by Zhang et al.^33^ Using a dataset of 23 patients, high CD36 expression had shorter, but not statistically significant, overall survival. Nevertheless, analyzing a dataset of 173 patients, those with high APOC2 or CD36 levels had significantly shorter overall survival than patients with low APOC2 and low CD36 expressions.^33^

A connection between CD36 expression and the tumor immune microenvironment is suggested. CD36 expression is significantly positively correlated with infiltrating stromal scores in AML, and there are positive correlations between CD36 expression and infiltrating levels of immune score in AML, as well as infiltrating levels of the Estimation of STromal and Immune cells in MAlignant Tumor tissues using Expression data (ESTIMATE) score in AML.^27^ Additionally, negative correlations were observed between CD36 expression and four methyltransferases in AML.^27^

CD36 expression was more frequently observed in an unfavorable cytogenetic group,^29^ suggesting a link between CD36 and high-risk cytogenetic profiles. Cytogenetic and molecular abnormalities appear to contribute to the severity driven by CD36 expression. CD36-positive patients with trisomy 8 had significantly poorer leukemia-free survival compared to CD36-positive patients without trisomy 8,^30^ highlighting the interplay between CD36 and specific cytogenetic alterations. Supporting this association, intermediate and unfavorable karyotypes exhibited a higher proportion of CD36-expressing blasts.^32^ CD36 expression was characterized as an independent marker for AML progression, but has a higher association in patients with *FLT3* abnormalities, reinforcing its role in poor prognosis.^32^ Moreover, AML cases in the CD36-high group were enriched with *KMT2A* (11q23) or t(9;22) abnormalities, while cases in the CD36-low group showed a higher frequency of favorable cytogenetic abnormalities, such as t(15;17) and t(8;21).^32^ This suggests that CD36 expression correlates with more aggressive disease phenotypes driven by adverse genetic features.

## Discussion

The results of this systematic review indicate that CD36 is associated with different prognosis factors in AML. For example, remission and relapse of the disease, leukemic cell metabolism and growth, overall survival, and chemoresistance.

There are some narrative reviews about the impact of CD36 on different types of cancer, which include AML, such as those reported by Wang et al.,^34^ Guerrero-Rodríguez et al.,^15^ and Feng et al.^35^ Some authors described studies that did not comply to the inclusion criteria, for instance, the study by Ye et al.^36^ demonstrated that leukemia stem cells with a higher CD36 expression seemed to be resistant to different chemotherapy drugs, such as cytarabine, doxorubicin, etoposide, SN38, and irinotecan. Since those were not systematic reviews, the authors used less strict criteria for inclusion, while this study aimed at demonstrating the correlation between CD36 and the prognosis of AML by evaluating clinical and not pre-clinical studies.

Some of the studies evaluated in this systematic review focus on children, associating a higher frequency of CD36-positive cells at diagnosis in children who experienced recurrence of AML^26^; therefore, studies comparing the role of CD36 in AML in different age ranges have not been done. Moreover, results from this research demonstrated that cells from AML patients expressing CD36 were less susceptible to chemotherapeutic agents such as Ara-C compared to cells that did not express CD36^28^; nevertheless, there are other drugs that must be investigated in further studies to support CD36 as a treatment target. Additionally, even though some studies focused on cytogenetic and molecular abnormalities in AML,^29^^,^^30^^,^^32^ this area is expanding and might be explored in further research to correlate the CD36 expression with different genetic features in AML patients.

The present study has some limitations. Many studies did not report or adjust the prognostic factor evaluated for other confounding factors and interventions that could affect the outcome. Also, some of the studies did not describe the characteristics of the population from which they obtained the bone marrow or peripheral blood samples. Moreover, the lack of data from the studies did not allow a meta-analysis. Therefore, further studies are necessary to empower evidence-building.

## Conclusions

In conclusion, this systematic review suggests that CD36 is associated with the prognosis of AML. The role of CD36 in the pathogenesis of AML remains to be evaluated to support CD36 as a treatment target.

## Funding

This work was supported by the Brazilian Coordination for the Improvement of Higher Education Personnel (Capes) finance code 001.

## Conflicts of interest

The authors declare no conflicts of interest.

## Appendix Supplementary materials

Image, application 1
